# Oncostatin M is a regulator of fibroblast growth factor 23 (FGF23) in UMR106 osteoblast-like cells

**DOI:** 10.1038/s41598-023-34858-6

**Published:** 2023-05-24

**Authors:** Sina Münz, Martina Feger, Michael Föller

**Affiliations:** Department of Physiology, University of Hohenheim, Stuttgart, Germany

**Keywords:** Physiology, Endocrinology, Medical research, Nephrology

## Abstract

Renal phosphate and vitamin D metabolism is under the control of fibroblast growth factor 23 (FGF23), an endocrine and paracrine factor predominantly produced in bone. FGF23 formation is stimulated by active vitamin D, or parathyroid hormone (PTH), which are further regulators of phosphate homeostasis. In renal, inflammatory, and other diseases, plasma FGF23 reflects disease stage and correlates with outcome. Oncostatin M is part of the interleukin-6 (IL-6) family and regulates remodeling and PTH effects in bone as well as cardiac FGF23 production in heart failure via glycoprotein gp130. Here, we studied whether oncostatin M is a regulator of FGF23 in bone cells. Experiments were performed in UMR106 osteoblast-like cells, *Fgf23* mRNA was determined by qRT-PCR, FGF23 protein by Western Blotting and ELISA, and oncostatin M receptor and leukemia inhibitory factor (LIF) receptor gene knockout accomplished by siRNA. As a result, oncostatin M dose-dependently up-regulated *Fgf23* expression and protein secretion. The oncostatin M effect on FGF23 was mediated by oncostatin M receptor and gp130 and involved, at least in part, STAT3 and MEK1/2. Taken together, oncostatin M is a regulator of FGF23 through oncostatin M receptor, gp130, as well as STAT3 and MEK1/2 in UMR106 osteoblasts.

## Introduction

The regulation of renal phosphate reabsorption is accomplished by bone hormone fibroblast growth factor 23 (FGF23)^1^ predominantly produced in osteocytes and osteoblasts^2^. FGF23 induces a decrease of surface expression of sodium/phosphate cotransporter NaPiIIa, a secondary-active electrogenic phosphate transporter expressed in the proximal tubule of the kidney^3,4^. Moreover, FGF23 is a potent regulator of vitamin D homeostasis, as it decreases the formation and induces the inactivation of 1,25(OH)_2_D_3_, the active form of vitamin D with hormone-like properties^5,6^. The effects of FGF23 in the kidney are dependent on a membrane receptor that comprises fibroblast growth factor receptor 1 (FGFR1) and transmembrane protein αKlotho^7,8^. In mice, lack of either FGF23 or αKlotho causes a severe derangement of phosphate and vitamin D metabolism ultimately resulting in a phenotype mimicking human aging with death at young age^8–10^. Apart from endocrine, bone-derived FGF23, other sources of FGF23 have been described including heart^11^, liver^12^, or kidney^13^. Extraosseous FGF23 may, at least in part, be effective under pathophysiological conditions and also induce paracrine effects^14–19^.

The mechanisms governing the production of FGF23 are of high interest since renal, cardiovascular, and further diseases have turned out to be associated with surges in plasma FGF23^20^. Especially in chronic kidney disease (CKD) and cardiovascular disease, FGF23 predicts outcome^21–23^. Moreover, FGF23 is involved in hepatic inflammation^12^ or left ventricular hypertrophy ^24^ and may, therefore, not only reveal disease activity, but also contribute to it.

Oncostatin M is a cytokine produced by leukocytes, but also by osteoblasts and osteocytes in bone and belongs to the interleukin-6 (IL-6) family^25,26^. Similar to other members of this family, oncostatin M signaling is dependent on glycoprotein gp130^27^. Osteoblasts and osteocytes are the very cells accounting for bone production of FGF23^28^. Inflammation has been demonstrated to up-regulate FGF23 production^29^, and IL-6 is among a group of pro-inflammatory cytokines contributing to this up-regulation^30^. Oncostatin M participates in bone remodeling^31^. It induces receptor activator of NF-κB ligand (RANKL), thereby enhancing osteoclast activity^32^. Parathyroid hormone (PTH) is a main stimulator of FGF23 production by acting on osteoblasts^33^, and it has been demonstrated to modulate oncostatin M signaling in these cells^34^. Importantly, in heart failure, oncostatin M stimulates cardiac FGF23 production^35^. However, to our knowledge, nothing is known about a oncostatin M effect on FGF23 production in bone.

Given the broad role of oncostatin M in osteoblast physiology and the fact, that IL-6 is a potent stimulator of FGF23 formation, we hypothesized that oncostatin M directly influences FGF23 through gp130 in bone cells. To test this hypothesis, we performed experiments in UMR106 osteoblast-like cells^36^ and explored the underlying mechanism.

## Results

**Figure 1:**
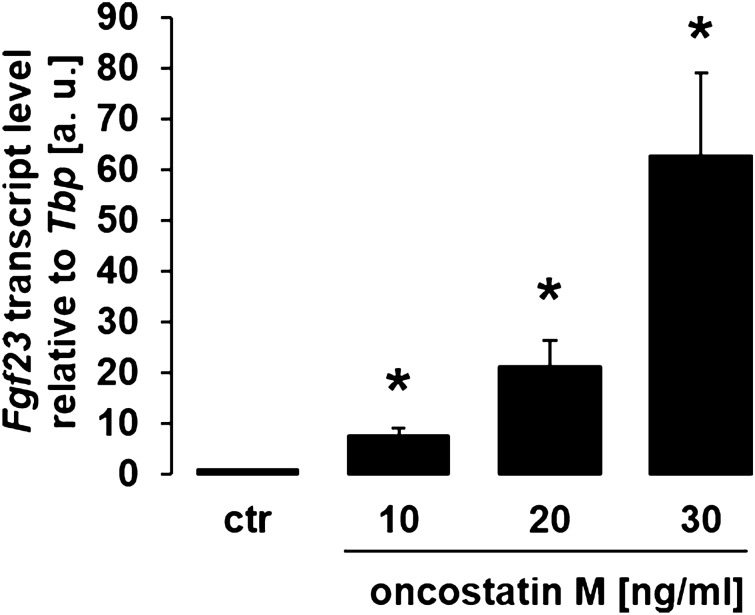
Oncostatin M up-regulated *Fgf23* expression in UMR106 cells in a dose-dependent manner. Arithmetic means ± SEM of *Fgf23* mRNA abundance relative to *Tbp* in osteoblast-like UMR106 cells treated without (ctr) or with the indicated concentrations of oncostatin M for 24 h (n = 5; one-sample *t* test). *p < 0.05 indicates significant difference from vehicle control. a. u. arbitrary units; ctr control.

To study whether bone FGF23 production is influenced by oncostatin M we used osteoblast-like UMR106 cells as a model. We incubated these cells with different concentrations of oncostatin M for 24 h and employed qRT-PCR to assess the consequence for *Fgf23* gene expression. We found a clear dose–response relationship for the oncostatin M effect on *Fgf23* mRNA abundance, pointing to the stimulation of *Fgf23* gene expression by oncostatin M in UMR106 osteoblasts (Fig. 1). In this series of experiments, housekeeping gene TATA box binding protein (*Tbp)* expression was not significantly affected by oncostatin M treatment (cycle threshold (Ct) value for *Tbp* ± SEM: 21.3 ± 0.21 in cells treated with 30 ng/ml oncostatin M versus 21.0 ± 0.12 in control cells; n = 5 for each, *p* > 0.05, paired *t* test).

**Figure 2:**
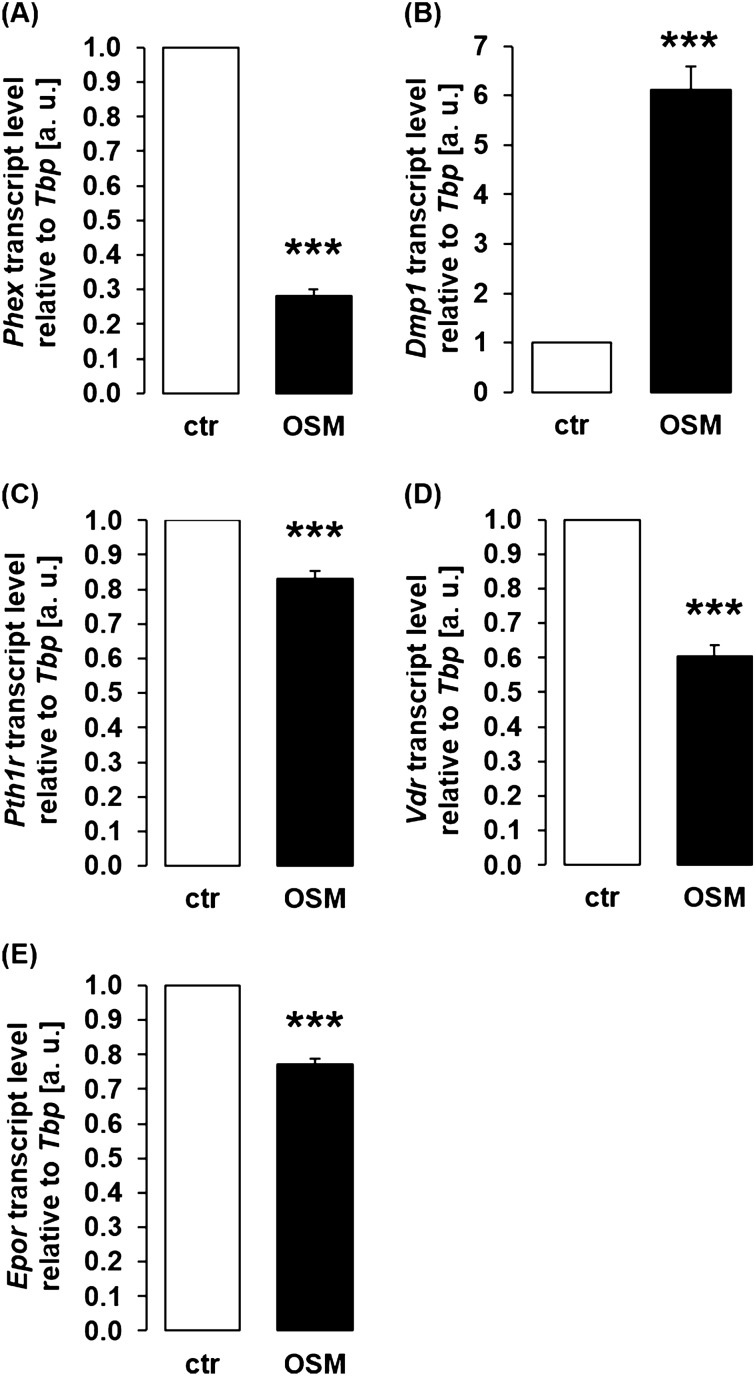
Oncostatin M affected FGF23 regulators in UMR106 cells. Arithmetic means ± SEM (n = 8) of *Phex* (**A**), *Dmp1* (**B**), *Pth1r* (**C**), *Vdr* (**D**), and *Epor* (**E**) mRNA expression relative to *Tbp* in UMR106 cells treated with or without 30 ng/ml oncostatin M for 24 h (one-sample *t* test). *** *p* < 0.001 indicates significant difference from vehicle control. a. u. arbitrary units; ctr control; Dmp1 dentin matrix acidic phosphoprotein 1; Epor erythropoietin receptor; OSM oncostatin M; Phex phosphate regulating endopeptidase X-linked; Pth1r PTH receptor; Vdr vitamin d receptor.

Since oncostatin M turned out to be an enhancer of *Fgf23* gene expression, we studied next whether oncostatin M impacts important regulators of FGF23. To this end, we assessed gene expression of bone proteins phosphate regulating endopeptidase X-linked (*Phex*) and dentin matrix acidic phosphoprotein (*Dmp1*), negative regulators of FGF23^37,38^. Whereas treatment of UMR106 cells with oncostatin M resulted in statistically significant down-regulation of *Phex* (Fig. 2A), *Dmp1* gene expression was significantly up-regulated by oncostatin M (Fig. 2B). Apart from these bone proteins, hormones including PTH, 1,25(OH)_2_D_3_, and erythropoietin (EPO) control *Fgf23* gene expression through their respective receptors^39–41^. Using qRT-PCR, we therefore analyzed whether oncostatin M alters gene expression of these receptors. As demonstrated in Fig. 2C–E, oncostatin M significantly down-regulated *Pth1r* (encoding the PTH receptor), *Vdr* (encoding the vitamin D receptor), and *Epor* (encoding the EPO receptor) gene expression in UMR106 cells.

**Figure 3:**
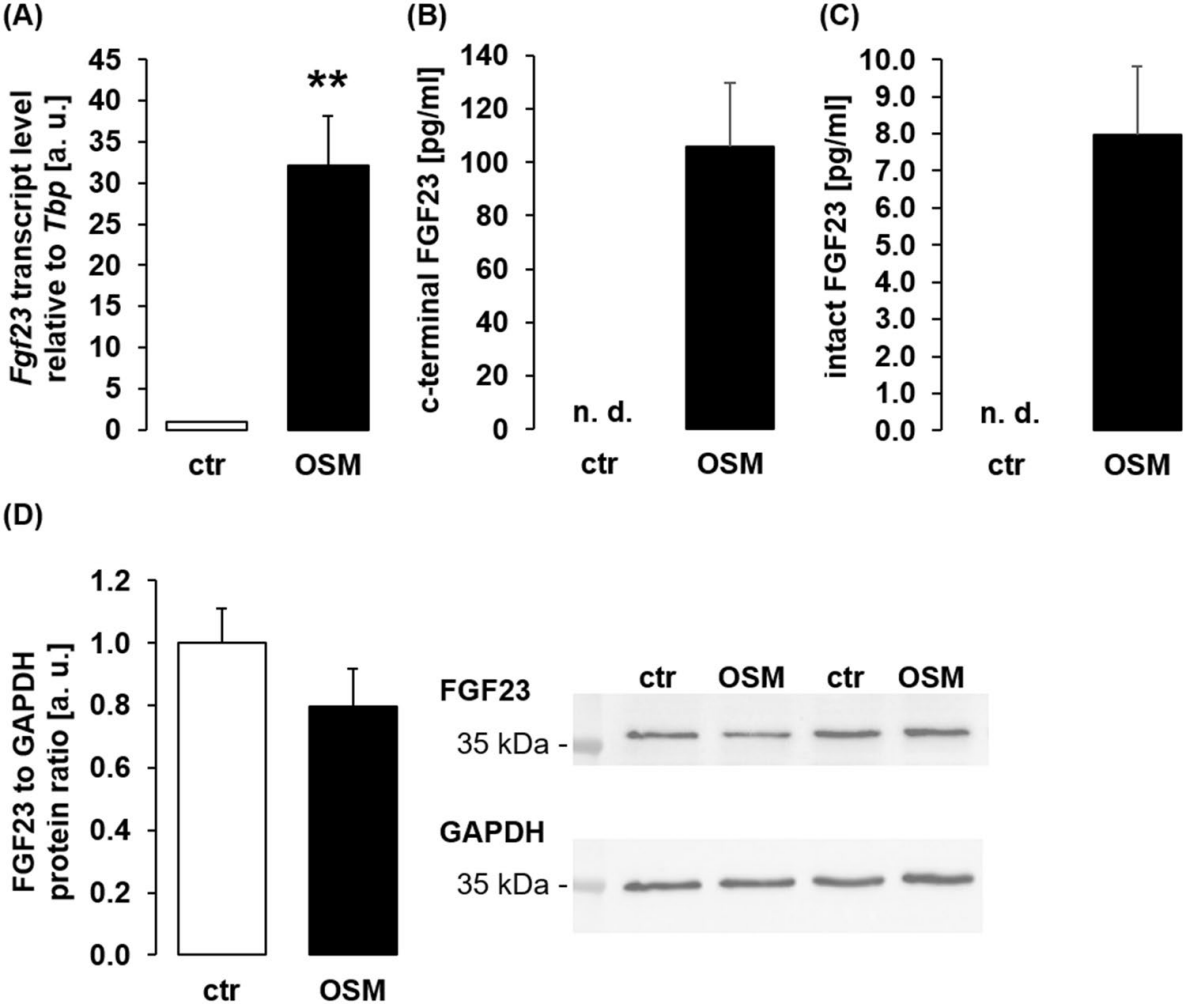
Oncostatin M induced FGF23 protein secretion by UMR106 cells. (**A**) Arithmetic means ± SEM of *Fgf23* mRNA levels relative to *Tbp* in UMR106 cells pretreated for 24 h with 10 nM PTH and then additionally treated with or without 100 ng/ml oncostatin M for another 24 h (n = 6; one-sample *t* test). Arithmetic means ± SEM of C-terminal (**B**; n = 5) and intact (**C**; n = 5) FGF23 concentration in the supernatant of UMR106 cells pretreated for 24 h with 10 nM PTH and then additionally treated with or without 100 ng/ml oncostatin M for another 24 h (n = 5; paired *t* test). (**D**) Representative original Western blots and arithmetic means ± SEM of normalized FGF23 over GAPDH protein ratio in lysates of UMR106 cells pretreated for 24 h with 10 nM PTH and then treated with or without 100 ng/ml oncostatin M for another 24 h (n = 5; paired *t* test). ** *p* < 0.01 indicates significant difference from vehicle control. a. u. arbitrary units; ctr control; GAPDH glyceraldehyde-3-phosphate dehydrogenase; n. d. not detectable; OSM oncostatin M.

The next series of experiments were performed to elucidate whether oncostatin M was also capable of modifying FGF23 protein secretion by UMR106 cells. To this end, cells were pretreated with PTH (10 nM, 24 h), which ramps up FGF23 production^42^ to a level enabling ELISA-based FGF23 quantifcation. As demonstrated in Fig. 3A, also in UMR106 cells pretreated with PTH, oncostatin M up-regulated *Fgf23* expression. Next, we used ELISA to measure FGF23 protein to investigate whether enhanced *Fgf23* gene expression is translated into protein synthesis. As illustrated in Fig. 3B and C, a 24-h oncostatin M treatment of UMR106 cells pre-stimulated with PTH strongly elevated C-terminal (Fig. 3B) and intact (Fig. 3C) FGF23 concentration in the cell culture supernatant, suggesting that oncostatin M indeed stimulated FGF23 production in bone cells. Next, we employed Western Blotting to determine FGF23 in the cell lysate. As demonstrated in Fig. 3D, cellular FGF23 protein expression was not significantly different between control cells and cells treated with oncostatin M.

**Figure 4:**
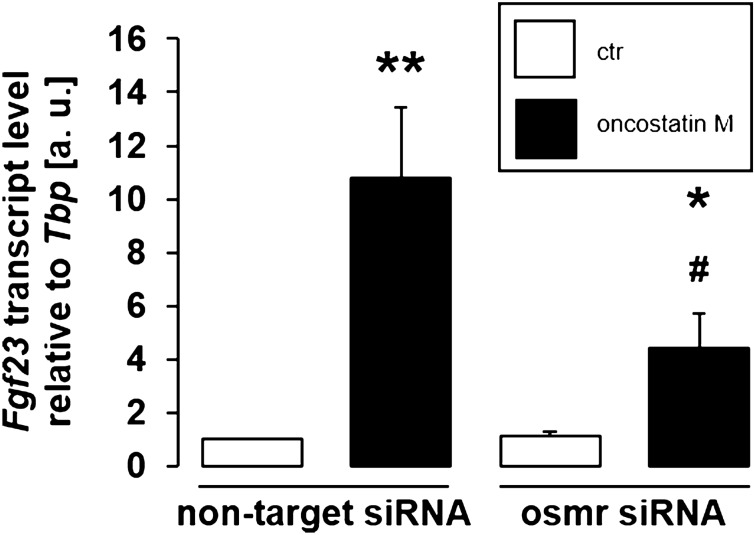
Oncostatin M receptor knockdown attenuated oncostatin M-dependent *Fgf23* gene expression. Arithmetic means ± SEM of *Fgf23* expression relative to *Tbp* in UMR106 cells treated for 24 h with or without 10 ng/ml oncostatin M in the presence of non-target siRNA (left bars) or siRNA specifically targeting the oncostatin M receptor (osmr) (right bars) (n = 11; Wilcoxon one-sample test and paired *t* test with Bonferroni adjustment for multiple comparisons). * *p* < 0.05, ** *p* < 0.01 indicate significant difference from vehicle control (1st bar); # *p* < 0.05 indicates significant difference from the absence of siRNA targeting the oncostatin M receptor (2nd bar vs. 4th bar). a. u. arbitrary units; ctr control; osmr oncostatin M receptor.

In order to study whether oncostatin M influences FGF23 through the oncostatin M receptor, we took benefit from siRNA-mediated knockdown. We treated UMR106 cells with or without 10 ng/ml oncostatin M for 24 h in the presence of non-target siRNA or siRNA specifically targeting the oncostatin M receptor. According to Fig. 4, silencing of the oncostatin M receptor significantly attenuated the oncostatin M effect on *Fgf23*. Silencing efficiency was 65 ± 5% (n = 4). Therefore, oncostatin M depends on the oncostatin M receptor to impact FGF23 in UMR106 cells.

Some extracellular signals of oncostatin M are mediated by the LIF receptor^25,43^. To study whether this receptor also accounts for oncostatin M-mediated stimulation of *Fgf23* gene expression, we employed RNA interference again. As demonstrated in suppl. Fig. 1, the oncostatin M effect on *Fgf23* gene expression was not significantly different between either UMR106 cells treated with 50 nM (suppl. Fig. 1A) or 100 nM (suppl. Fig. 1B) siRNA specifically targeting the LIF receptor on the one hand and treated with the respective amount of non-target siRNA on the other hand. Efficiency was 32 ± 3% (n = 4) upon silencing with 50 nM siRNA and 42 ± 4% (n = 4) when using 100 nM siRNA.

**Figure 5:**
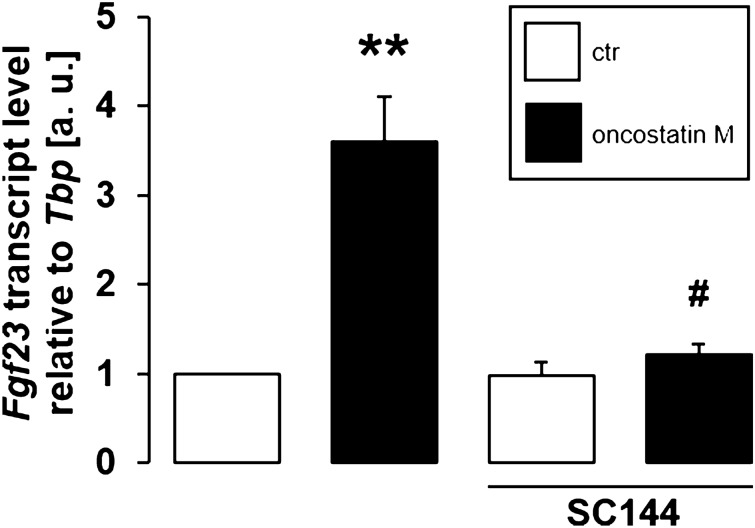
Gp130 inhibitor SC144 blunted oncostatin M-dependent *Fgf23* gene expression. Arithmetic means ± SEM of *Fgf23* mRNA abundance relative to *Tbp* in UMR106 cells treated with or without 30 ng/ml oncostatin M for 24 h in the presence or absence of 1 µM gp130 inhibitor SC144 (n = 7; one-sample *t* test and Wilcoxon signed-rank test with Bonferroni adjustment for multiple comparisons). ** *p* < 0.01 indicates significant difference from vehicle control (1st bar); # *p* < 0.05 indicates significant difference from the absence of SC144 (2nd bar vs. 4th bar). a. u. arbitrary units; ctr control.

Further experiments addressed the signaling of oncostatin M-dependent stimulation of FGF23 in UMR106 cells. Similar to FGF23 inducer IL-6, oncostatin M effects are mediated by gp130^27^. We treated UMR106 cells with and without oncostatin M (30 ng/ml) in the presence or absence of gp130 inhibitor SC144 (1 µM) for 24 h. As shown in Fig. 5, the oncostatin M effect on *Fgf23* gene expression was significantly blunted by SC144. Hence, gp130 was required for oncostatin M to up-regulate FGF23.

**Figure 6:**
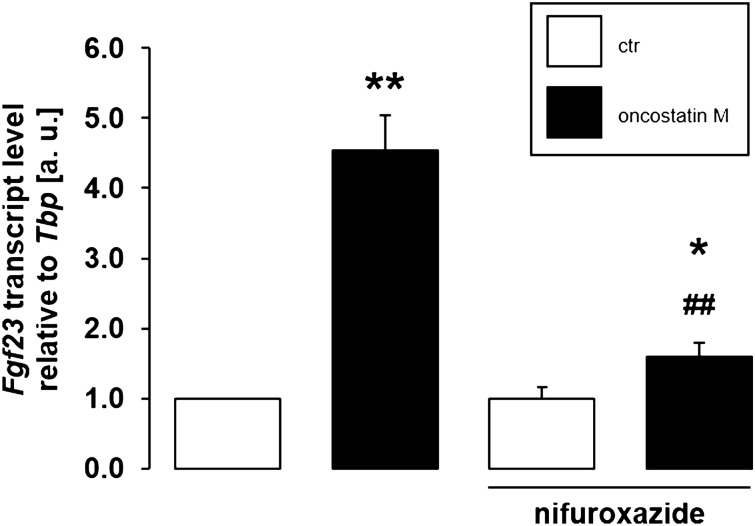
STAT3 inhibitor nifuroxazide reduced oncostatin M-dependent *Fgf23* gene expression. Arithmetic means ± SEM of *Fgf23* mRNA abundance relative to *Tbp* in UMR106 cells treated with or without 10 ng/ml oncostatin M for 24 h in the presence or absence of 10 µM STAT3 inhbitor nifuroxazide (n = 6; one-sample *t* test and paired *t* test with Bonferroni adjustment for multiple comparisons). * *p* < 0.05, ** *p* < 0.01 indicate significant difference from vehicle control (1st bar); ## *p* < 0.01 indicates significant difference from the absence of nifuroxazide (2nd bar vs. 4th bar). a. u. arbitrary units; ctr control.

Further downstream signaling of oncostatin M may involve transcription factor STAT3^44^. To explore its involvement, we utilized STAT3 inhibitor nifuroxazide. It is illustrated in Fig. 6 that oncostatin M-induced up-regulation of *Fgf23* was significantly attenuated by 10 µM nifuroxazide, pointing to STAT3 being required for oncostation M to enhance *Fgf23* gene expression.

**Figure 7:**
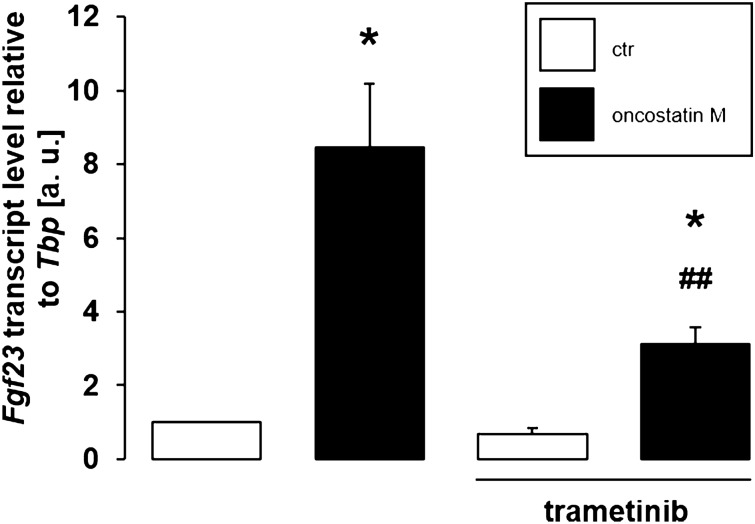
MEK1/2 inhibitor trametinib attenuated oncostatin M-dependent *Fgf23* gene expression. Arithmetic means ± SEM of *Fgf23* mRNA abundance relative to *Tbp* in UMR106 cells treated with or without 10 ng/ml oncostatin M for 24 h in the presence or absence of 10 µM MEK1/2 inhibitor trametinib (n = 5; one-sample *t* test and paired *t* test with Bonferroni adjustment for multiple comparisons). * *p* < 0.05 indicates significant difference from vehicle control (1st bar); ## *p* < 0.01 indicates significant difference from the absence of trametinib (2nd bar vs. 4th bar). a. u. arbitrary units; ctr control.

Alternatively, oncostatin M may ultimately induce mitogen-activated extracellular signal-regulated kinases 1 und 2 (MEK1/2) activity^45^. We carried out further experiments with trametinib, a MEK1/2 inhibitor, to elucidate whether these kinases also contribute to the oncostatin M effect on *Fgf23*. As displayed in Fig. 7, the oncostatin M effect on *Fgf23* was significantly lower in the presence of trametinib, suggesting that MEK1/2 also mediate oncostatin M-dependent up-regulation of *Fgf23* gene expression.

Since PTH enhances FGF23 production in a protein kinase A (PKA)-dependent manner^41^, we determined phospho-PKA-C (pPKA-C) protein abundance in UMR106 cells by Western Blotting. As demonstrated in suppl Fig. 2A, treatment with oncostatin M did not significantly modify pPKA-C protein abundance. Neither did PTH treatment (suppl. Fig. 2B).

Lastly, we bred mice expressing oncostatin M receptors in all tissues and organs but bone osteocytes (Osmr_osteocyte_^−/−^). C-terminal FGF23 serum levels tended to be lower in Osmr_osteocyte_^−/−^ mice (318.2 ± 33.4 pg/ml; n = 6) compared to control animals Osmr_osteocyte_^+/+^ mice (374.7 ± 76.1 pg/ml; n = 6). The difference did, however, not reach statistical significance.

## Discussion

According to our study, oncostatin M is a stimulator of FGF23 production in UMR106 osteoblast-like cells. The oncostatin M effect was dependent on the oncostatin M receptor and gp130. According to our silencing experiment, however, LIF receptor, which is reported to mediate some of the cellular effects of oncostatin M^25,43^, did not contribute to the up-regulation of *Fgf23* transcripts induced by oncostatin M.

We uncovered that oncostatin M ramped up Fgf23 gene expression and the FGF23 protein concentration in the cell culture supernatant, in line with a stimulatory effect of oncostatin M on FGF23 production. That cellular FGF23 protein levels were not significantly different between control cells and cells exposed to oncostatin M may point to the vast part of FGF23 protein being secreted in response to oncostatin M.

Oncostatin M is part of the IL-6 family of cytokines^46^. Signaling pathways within this family typically involve gp130, a transmembrane glycoprotein^47^. Our experiments with gp130 inhibitor SC144 uncovered that also oncostatin M-dependent up-regulation of FGF23 in UMR106 cells requires gp130. Further downstream signaling was, at least in part, dependent on STAT3 and MEK1/2 as experiments with inhibitors nifuroxazide and trametinib revealed. According to Western Blotting PKA was rather not involved in the oncostatin M effect on FGF23. Although PTH induces PKA activity, we did not observe a significant effect of PTH on pPKA-C protein levels. It must kept in mind that cells were cultured in the presence of serum and hence several factors which can be expected to impact PKA activity. Moreover, PKA phosphorylation is an early event whereas our Western Blot analysis was carried out after prolonged incubation.

Chronic kidney disease (CKD) is characterized by a very early rise in plasma FGF23, which is classically attributed to hyperphosphatemia and hyperparathyroidism^48^. However, also inflammation is effective in stimulating FGF23 production in CKD^49^, and, in a broader sense, inflammation is an important trigger of FGF23 production^29^. Notably, pro-inflammatory cytokines including IL-6 have been shown to directly induce FGF23^30^. Therefore, the stimulation of FGF23 synthesis by oncostatin M is in line with these observations and may be relevant under inflammatory conditions that result in elevated oncostatin M levels, including inflammatory bowel disease^50^, but may also play a role in cancer^51^. Juvenile inflammatory bowel disease^52^ and different forms of cancer^53^ are indeed associated with higher FGF23 plasma levels. It is therefore tempting to speculate that oncostatin M elevations in these disorders and further diseases may directly contribute to enhanced FGF23 production.

Moreover, oncostatin M is involved in the acute phase reaction in deceased donor kidneys^54^, and also in obstructive nephropathy, renal oncostatin M production is increased^55^. Hence, increased renal production of oncostatin M may also contribute to the enhanced FGF23 production characteristic of kidney disease.

A clear role of oncostatin M in bone physiology has already been demonstrated^56^. Oncostatin M is involved in the PTH effect on bone^34^, and fosters bone formation^25^ and healing^57^. Our findings, i.e. the stimulation of FGF23 production by UMR106 osteoblasts, fits well into the concept of oncostatin M being an important regulator of bone physiology, too.

Heart failure is a medical condition that can also be the typical sequela of higher stage CKD^58^. It is associated with elevations in plasma FGF23 predicting outcome^59^. Oncostatin M has already been shown to increase FGF23 production by cardiomyocytes in heart failure^35^. Our study suggests that bone may also be a source of FGF23 independent of heart failure, e.g. in inflammatory conditions associated with enhanced production of oncostatin M.

Oncostatin M had complex effects on established regulators of FGF23 in bone cells. Whereas Phex expression was suppressed by oncostatin M, Dmp1 expression went up although both, Phex and Dmp1 are negative bone regulators of FGF23. It appears to be possible that oncostatin M-dependent down-regulation of Phex was required for the up-regulation of FGF23, and that up-regulation of Dmp1 is a compensatory mechanism in response to higher FGF23 production. Alternatively, Phex and Dmp1 are regulated by oncostatin M in an FGF23-independent manner. The downregulation of the expression of the genes encoding the PTH, 1,25(OH)_2_D_3_, and EPO receptor may be interpreted as a compensation for oncostatin M-dependent up-regulation of FGF23 as the three hormones are inducers of FGF23. However, FGF23-independent effects of oncostatin M on the receptor proteins may also be relevant as EPO activates the OSM promoter^60^, and PTH also utilizes oncostatin M signaling^34^. Our study does not allow safe conclusion as to whether the observed changes in gene expression of the FGF23 regulators are coincidental or causative for the oncostatin M effect on FGF23.

In order to estimate the in vivo relevance of oncostatin M for the regulation of FGF23, we bred mice with specific deletion of the oncostatin M receptor in osteocytes. Whereas the C-terminal FGF23 serum concentration tended to be lower in these animals compared to control mice, the effect was not statistically significant. Therefore, lack of oncostatin M receptor signaling may be compensated in vivo by other stimulators of FGF23. Otherwise, oncostatin M receptor may only be relevant under certain pathophysiological conditions. Clearly, more investigations into the precise in vivo role of oncostatin M are warranted.

In conclusion, we revealed oncostatin M, a member of the IL-6 family of cytokines, as an inducer of FGF23 production in UMR106 osteoblast-like cells, an effect involving oncostatin M receptor and gp130 but not LIF receptor. Oncostatin M-dependent up-regulation of FGF23 may be relevant in different diseases associated with enhanced oncostatin M levels and was, at least in part, dependent on intracellular STAT3 and MEK1/2 signaling.

## Materials and methods

### Cell culture

Rat osteoblast-like UMR106 cells (CRL-1661; ATCC, Manassas, VA, USA) were grown at 5% CO_2_ and 37 °C in high glucose Dulbecco’s Modified Eagle Medium (DMEM) (Gibco, Life Technologies, Thermo Fisher Scientific, Darmstadt, Germany) supplemented with 10% fetal bovine serum (FBS; Gibco, Life Technologies), 100 U/ml penicillin, and 100 µg/ml streptomycin (Gibco, Life Technologies). Cells were seeded into 6-well plates (Greiner Bio-One, Frickenhausen, Germany) at a density of 200,000 cells per well, and after 24 h, recombinant rat oncostatin M (Peprotech Hamburg, Germany) was added for another 24 h. Gp130 inhibitor SC144 (Bio-Techne, Tocris, Bristol, UK) was used at 1 µM for 24 h, STAT3 inhibitor nifuroxazide (Merck, Damstadt, Germany) at 10 µM for 24 h, and MEK1/2 inhibitor trametinib (Biomol, Hamburg, Germany) at 10 µM for 24 h. Control cells were treated with the appropriate amount of vehicle. When unstimulated, FGF23 protein expression is low in UMR106 cells ^61^. In order to ramp up expression to a level above the ELISA detection limit, 10 nM PTH (Merck, Darmstadt, Germany) was added 24 h prior to oncostatin M treatment.

### Small interfering RNA (siRNA)

UMR106 cells were transfected in antibiotic-free cell culture medium using transfection reagent DharmaFECT 1 (Dharmacon, Lafayette, CO, USA) and 25 nM siRNA targeting oncostatin M receptor (LQ-087727-02; Dharmacon), or 50 nM or 100 nM siRNA targeting LIF receptor (L-092591-02; Dharmacon), or the respective amount of non-target siRNA (D-001810-10; Dharmacon) as negative control, respectively. Cells were transfected 24 h prior to oncostatin M treatment.

### Quantitative real time PCR (qRT-PCR)

RNA was isolated from UMR106 cells using peqGOLD TriFast reagent (VWR, Bruchsal, Germany), and 1.2 µg of total RNA (60 ng/µl) was used for cDNA synthesis with the GoScript reverse transcription system and random primers (both from Promega, Mannheim, Germany). For analysis of erythropoietin receptor (*Epor*) transcript levels, isolated RNA was treated with DNase (Thermo Fisher Scientific) before cDNA synthesis. The quantitative real time PCR (qRT-PCR) analyses were carried out with a CFX Connect Real-Time PCR System (Bio-Rad Laboratories, Feldkirchen, Germany). The reaction master mix contained 2 µl cDNA, 0.5 µM (or 0.25 µM for *Fgf23*, *Vdr*, and *Lifr*) forward and reverse primer, 10 µl GoTaq qPCR Master Mix (Promega), and sterile water up to 20 µl.

The following rat primers were used (5′ → 3′):

*Fgf23*: TAGAGCCTATTCAGACACTTC and CATCAGGGCACTGTAGATAG; *Tbp*: ACTCCTGCCACACCAGCC and GGTCAAGTTTACAGCCAAGATTCA; *Phex*: ATGGCTGGATAAGCAATAAC and GCTTTTTCAATCGCTTTCTC; *Dmp1*: CGCCCATGGCAAATAGTGAC and CGTGCTGTCTTCACTGGACT; *Pth1r*: GAAGTTCTGCACACAGCAGC and ATGCCTTCTCTTTCCTGGGC; *Vdr*: ATGAAGGAGTTCATCCTGAC and ATGATGTGCTGTTGTTCTTC; *Epor*: AGGTGGACGTGTCAGCAGGC and CCCCGCAGGTTGCTCAGGAC; *Osmr*: GAAGGAGAAGTGATACATGAG and AGTCACTCCATTTCCAGAAG; *Lifr*: AAGAGTTGCTCTTCATGTTC and ACATGGTAGGTTGAATCCTC.

Transcript levels were normalized to transcript levels of housekeeping gene *Tbp*, and analyzed with the 2^−ΔΔCt^ method.

### Measurement of FGF23 protein in cell culture supernatant

Cell culture supernatants were collected and concentrated using Vivaspin 6 ultrafiltration columns (Sartorius, Göttingen, Germany). C-terminal and intact FGF23 protein concentration was measured by enzyme-linked immunosorbent assay (ELISA) according to the manufacturer’s protocol (both from Immutopics, San Clemente, CA, USA).

### Western blotting

UMR106 cells treated with or without 100 ng/ml oncostatin M for 24 h were lysed in ice-cold RIPA buffer (Cell signaling, Frankfurt, Germany) supplemented with complete protease and phosphatase inhibitor cocktail and EDTA (Thermo Fisher Scientific). After centrifugation at 10,000 g and 4 °C for 5 min, proteins were boiled in Roti-Load 1 buffer (Carl Roth, Karlsruhe, Germany) for 10 min. Proteins (30 µg per lane) were separated on 12% SDS polyacrylamide gels, and transferred to nitrocellulose membranes. Membranes were incubated overnight at 4 °C with rabbit anti-FGF23 antibody (diluted 1:1000, #BS-5768R; Thermo Fisher Scientific) or rabbit anti-GAPDH antibody (diluted 1:2000, #5174; Cell Signaling), and then with secondary goat anti-rabbit HRP-conjugated antibody (1:5000; Cell Signaling) for 1 h at room temperature. For loading control, membranes were stripped in stripping buffer (Roti-Free Stripping buffer 2.2 plus; Carl Roth, Karlsruhe, Germany) at room temperature for 30 min. Antibody binding was detected with ECL detection reagent (Bio-Rad Laboratories) and densitometric analysis was performed by using Image Lab software 6.1 (Bio-Rad Laboratories). The results are shown as the ratio of FGF23 protein to GAPDH, normalized to the control group.

### Animals

Osteocyte-specific oncostatin M receptor (Osmr) knock-out mice (Osmr^fl//fl^ Dmp1-Cre, in the following referred to as Osmr_osteocyte_^-/-^) were generated by crossing B6;129-*Osmr*^*tm1.1Nat*^/J mice (#011081; The Jackson Laboratory, Bar Harbor, ME, USA) and B6N.FVB-Tg(Dmp1-cre)1Jqfe/BwdJ mice (#023047; The Jackson Laboratory). Osmr^fl/fl^ mice have loxP sites on either side of the second exon (first coding exon) in the *Osmr* gene. The Cre-recombinase is under the control of the murine Dmp1 promoter/enhancer elements. At the age of 16 weeks, male and female mice were sacrificed, and serum concentration of C-terminal FGF23 was determined by ELISA (Immutopics). As controls (Osmr_osteocyte_^+/+^), mice with either Dmp1-Cre only or Osmr^fl/fl^ only were used.

### Statistics

Data are shown as arithmetic means ± standard error of the mean (SEM), *n* represents the number of independent experiments or mice per genotype. Normal distribution was tested with Shapiro–Wilk normality test. One-sample *t* test or one-sample Wilcoxon test if values were not normally distributed was used for comparisons with vehicle control. Other comparisons were made with paired *t* test or Wilcoxon-signed rank test, as indicated. Multiple comparisons were corrected with Bonferroni adjustment. Differences between control (Osmr_osteocyte_^+/+^) and Osmr_osteocyte_^−/−^ mice were assessed by two-tailed unpaired *t* test. Differences were considered significant if *p* < 0.05. IBM SPSS Statistics (Version 27.0; Armonk, NY, USA) was used.

### Ethics approval

All animal experimental protocols were approved by the animal protection officer of the University of Hohenheim (T 204/21). All methods were carried out in accordance with relevant guidelines and regulations and are reported in accordance with ARRIVE guidelines.

## Supplementary Information

Supplementary Information.

## Data Availability

All data are available from the corresponding author upon reasonable request.
