# Supplementary material for: Oncostatin M is a regulator of fibroblast growth factor 23 (FGF23) in UMR106 osteoblast-like cells
Source: Sci Rep. 2023 May 24;13:8420. doi: 10.1038/s41598-023-34858-6 (PMC10209182; doi:10.1038/s41598-023-34858-6)
Supplement: Supplementary file 1 — Supplementary Information. [file 41598_2023_34858_MOESM1_ESM.pdf]

Supplemental Material

Materials and methods

Western Blotting

Osteoblast-like UMR106 cells treated with or without 10 nM PTH for 48 h, and UMR106 cells treated with or without 100 ng/ml oncostatin M in the presence of PTH (10 nM; 48 h) for 24 h were lysed in ice-cold RIPA buffer (Cell signaling, Frankfurt, Germany) supplemented with complete protease and phosphatase inhibitor cocktail and EDTA (Thermo Fisher Scientific, Frankfurt, Germany). After centrifugation at 10,000 g and 4°C for 5 min, proteins were boiled in Roti-Load 1 buffer (Carl Roth, Karlsruhe, Germany). Proteins (30 µg per lane) were separated on 10% SDS polyacrylamide gels, and transferred to nitrocellulose membranes. Membranes were incubated overnight at 4°C with rabbit anti-phospho-PKA C (Thr197) antibody (diluted 1:2,000, #4781; Cell Signaling), and then with secondary goat anti-rabbit HRP-conjugated antibody (1:5,000; Cell Signaling) for 1 h at room temperature. For loading controls, membranes were stripped (Roti-Free Stripping buffer 2.2 plus; Carl Roth, Karlsruhe, Germany) at room temperature for 30 min, and then incubated with rabbit anti-GAPDH antibody (diluted 1:2,000, #5174; Cell Signaling). Bands were visualized with ECL detection reagent (Bio-Rad Laboratories, Feldkirchen, Germany) and densitometric analyses were conducted by using Image Lab software 6.1 (Bio-Rad Laboratories). The results are presented as the ratio of phospho-PKA C over GAPDH, normalized to the control group.

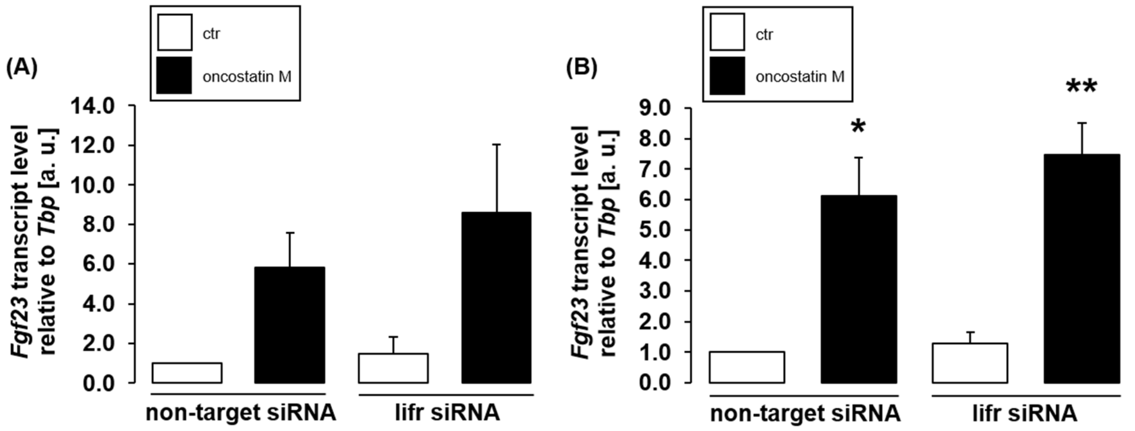

Suppl. Figure 1. Leukemia inhibitor factor (LIF) receptor knockdown did not significantly affect oncostatin M-dependent *Fgf23* gene expression.

(**A**,**B**) Arithmetic means ± SEM of *Fgf23* expression relative to *Tbp* in UMR106 cells treated for 24 h with or without 10 ng/ml oncostatin M in the presence of non-target siRNA (left bars) or siRNA specifically targeting LIF receptor (lifr) (right bars). 50 nM (**A**) or 100 nM (**B**) siRNA was used (**A**,**B**: n = 4; one-sample *t* test and paired *t* test with Bonferroni adjustment for multiple comparisons).

* $p < 0.05$, ** $p < 0.01$ indicate significant difference from vehicle control (1st bar).

a. u. arbitrary units; ctr control; lifr leukemia inhibitory factor receptor.

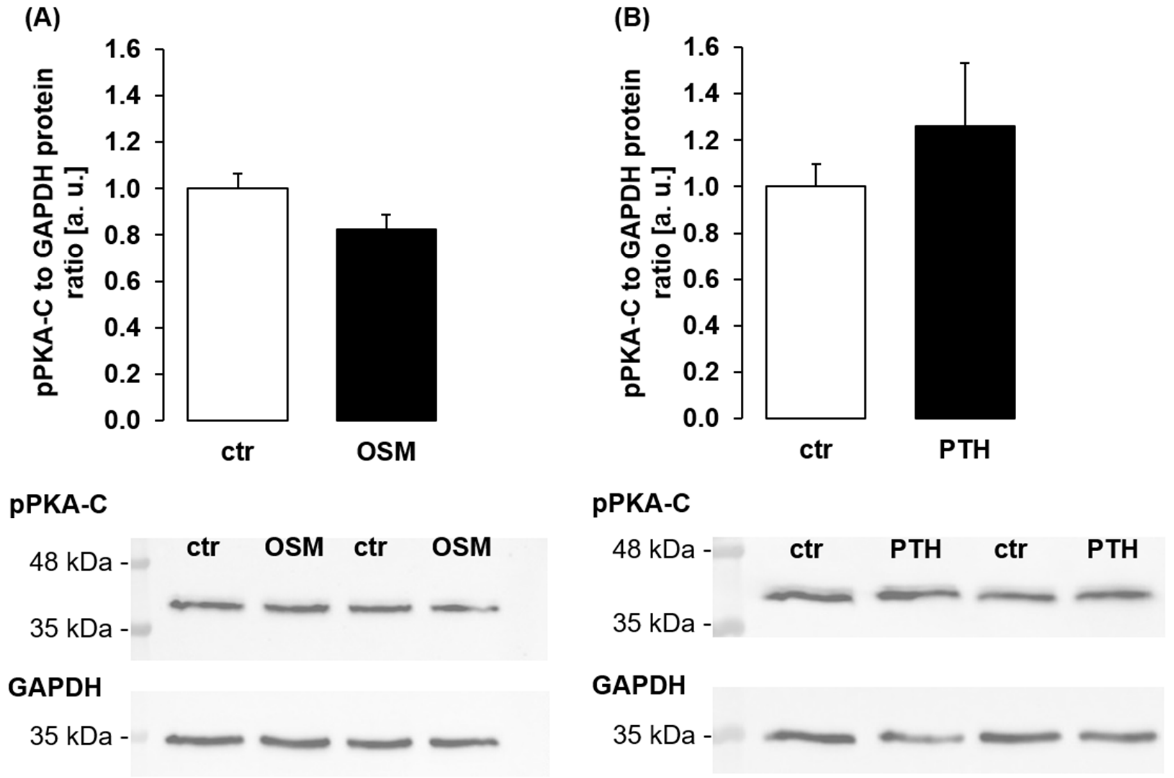

Suppl. Figure 2. Prolonged oncostatin M and PTH treatment did not significantly alter phospho-PKA-C abundance in UMR106 cells.

(**A**) Representative original Western blots and arithmetic means ± SEM of normalized phosphorylated PKA-C over GAPDH protein ratio in UMR106 cells treated with or without 100 ng/ml oncostatin M for 24 h in the presence of 10 nM PTH (48 h) (n = 5; paired *t* test).

(**B**) Representative original Western blots and arithmetic means ± SEM of normalized phosphorylated PKA-C to GAPDH protein ratio in UMR106 cells treated with or without 10 nM PTH for 48 h (n = 4; paired *t* test).

a. u. arbitrary units; ctr control; GAPDH glyceraldehyde-3-phosphate dehydrogenase; PKA-C cAMP-dependent protein kinase; pPKA-C phosphorylated PKA-C; PTH parathyroid hormone; OSM oncostatin M.

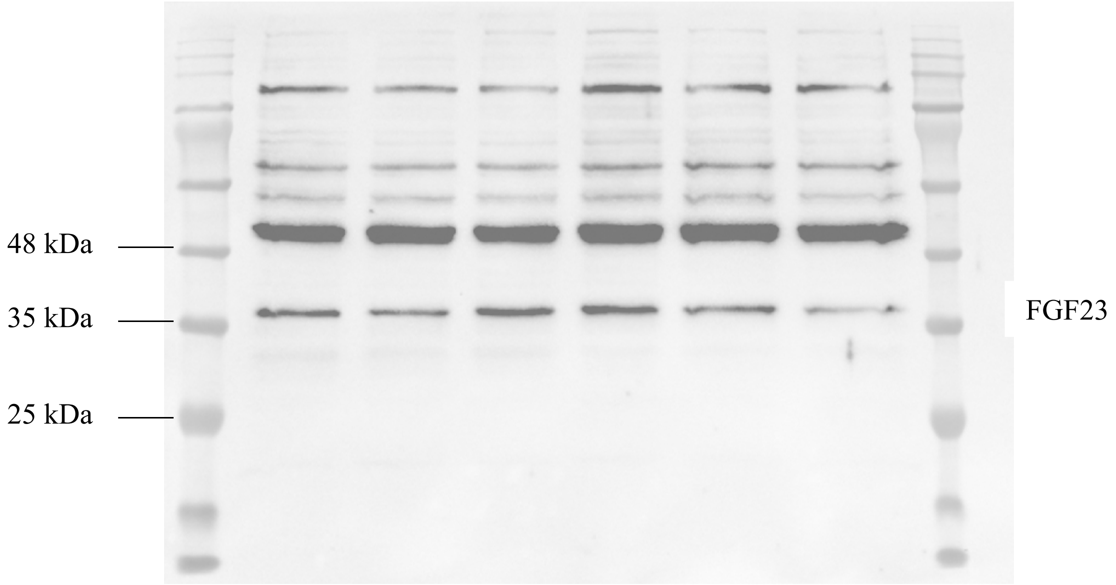

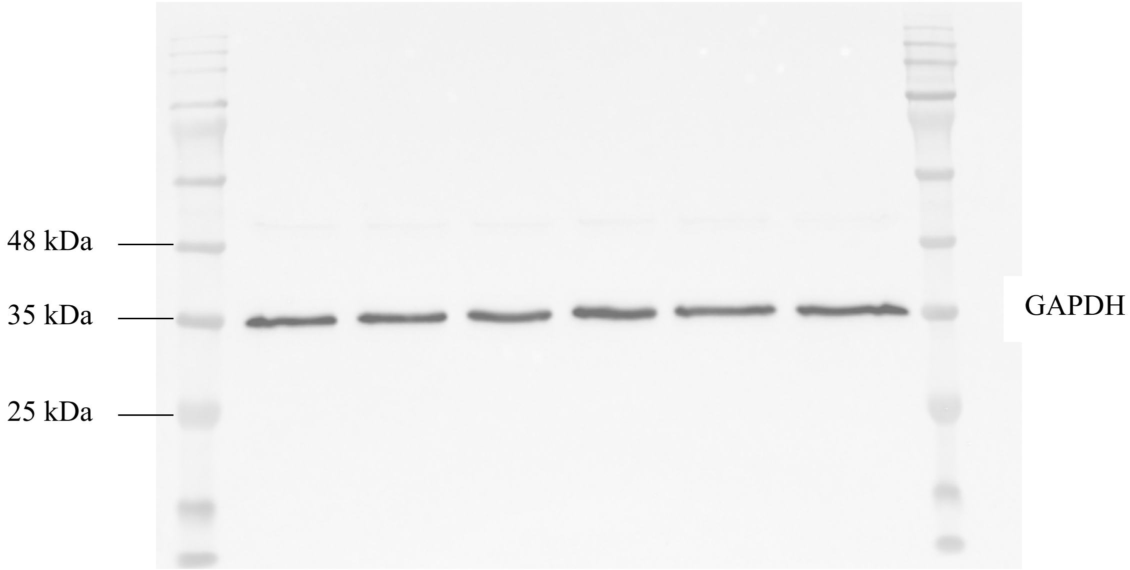

Full-length blots for Fig. 3D

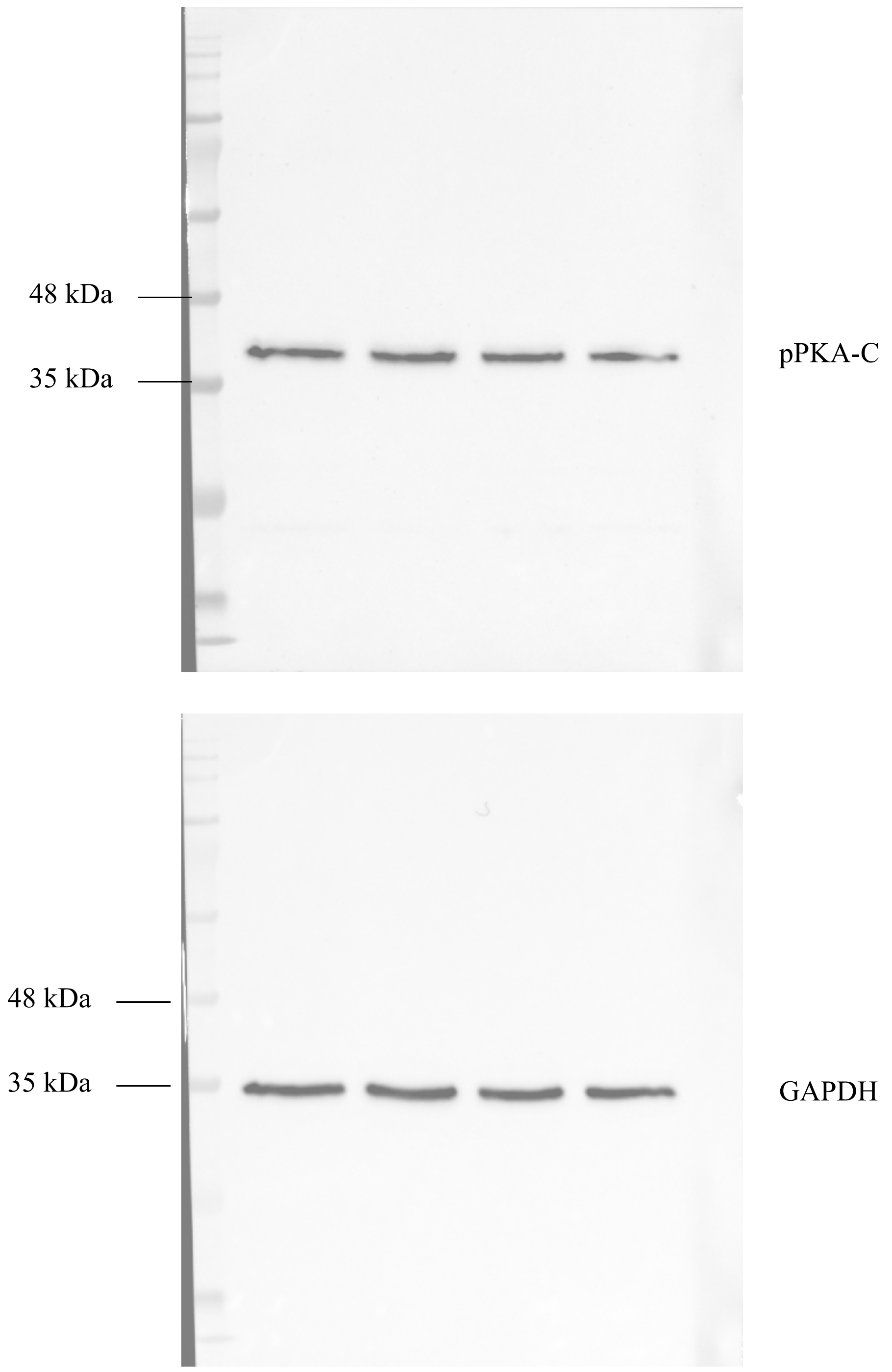

Full-length blots for suppl. Fig. 2A

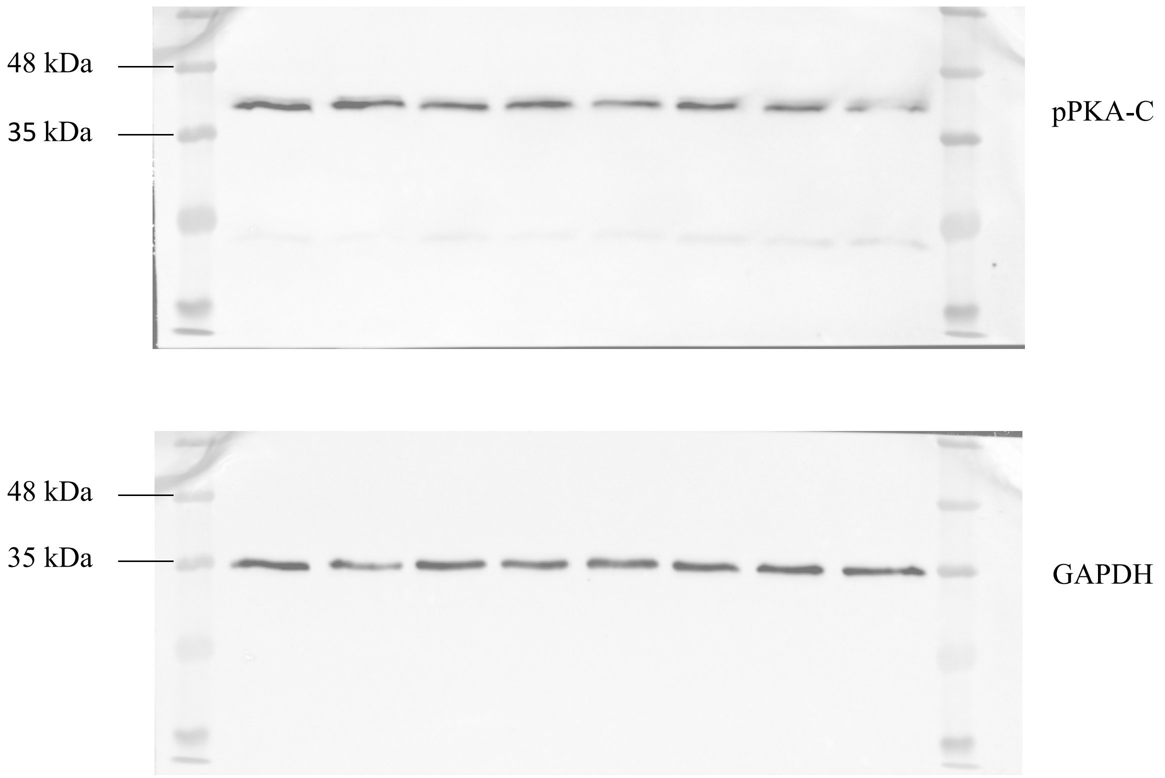

Full-length blots for suppl. Fig. 2B
